# To Extinguish the Fire from Outside the Cell or to Shutdown the Gas Valve Inside? Novel Trends in Anti-Inflammatory Therapies

**DOI:** 10.3390/ijms160921277

**Published:** 2015-09-07

**Authors:** Annalisa Marcuzzi, Elisa Piscianz, Erica Valencic, Lorenzo Monasta, Liza Vecchi Brumatti, Alberto Tommasini

**Affiliations:** 1 Department of Medicine, Surgery and Health Sciences, University of Trieste, Piazzale Europa 1, Trieste 34128, Italy; 2 Institute for Maternal and Child Health - IRCCS “Burlo Garofolo” - , via dell’Istria, 65/1, Trieste 34137, Italy; E-Mails: elisa.piscianz@gmail.com (E.P.); erica.valencic@gmail.com (E.V.); lorenzo.monasta@burlo.trieste.it (L.M.); liza.vecchibrumatti@burlo.trieste.it (L.V.B.); alberto.tommasini@burlo.trieste.it (A.T.)

**Keywords:** cytokines, small molecules, biologic drugs, rare disease

## Abstract

Cytokines are the most important soluble mediators of inflammation. Rare pediatric diseases provided exemplar conditions to study the anti-inflammatory efficacy of new generation therapies (biologics/biopharmaceuticals) selectively targeting single cytokines. Monoclonal antibodies and recombinant proteins have revolutionized anti-inflammatory therapies in the last two decades, allowing the specific targeting of single cytokines. They are very effective in extinguishing inflammation from outside the cell, even with the risk of an excessive and prolonged immunosuppression. Small molecules can enter the cell and shutdown the valve of inflammation by directly targeting signal proteins involved in cytokine release or in response to cytokines. They are orally-administrable drugs whose dosage can be easily adjusted to obtain the desired anti-inflammatory effect. This could make these drugs more suitable for a wide range of diseases as stroke, gout, or neurological impairment, where inflammatory activation plays a pivotal role as trigger. Autoinflammatory diseases, which have previously put anti-cytokine proteins in the limelight, can again provide a valuable model to measure the real potential of small inhibitors as anti-inflammatory agents.

## 1. Introduction

Cytokines are soluble mediators involved in signaling between different cells. They are particularly important in the immune system, but several cytokines can be produced by non-immune cells and can also act on non-immune tissues, such as bone, muscle, and endothelia [1,2]. The outcome of immune responses is greatly influenced by the set of cytokines produced, which can be reflected on the recruitment and activation of different effector cells in tissues and on different systemic effects.

Although the number of known cytokines is quite high, some of them seem to have a pivotal role in orchestrating the immune response thanks to their action on a high- controlled interaction network. In fact, complex clinical phenotypes are associated with deregulated secretion of few cytokines, such as interleukin (IL)-1, IL-6, tumor necrosis factor (TNF)-α, and Type 1 interferons (IFN). Conversely, selective inhibition of a single cytokine has resulted in a deeper anti-inflammatory action than that of traditional steroidal or non-steroidal anti-inflammatory drugs. However, patients treated with monoclonal antibodies directed against immunological molecules may display clinical features similar to patients with primary immunodeficiency (PID) of the corresponding antibody target [3], highlighting the risk of excessive immunosuppression.

Thus, the development of small molecules inhibiting cytokine signaling deserves much attention, not only for the ease of their oral administration, but also for the possibility to adjust the dosage for an optimal tuning of cytokine levels, or for the possibility of a prompt suspension of the treatment in cases of severe infections [4].

In the last decades, a number of rare monogenic diseases, with early onset in childhood, offered exemplary models to test the action of antibodies and other biological drugs targeting inflammatory cytokines. The same disorders can provide the ideal setting to study the action and the potential of novel molecules targeting cytokine signaling.

In this review, we will focus, in particular, on three examples: Cryopyrin-Associated Periodic Syndrome, an autoinflammatory diseases associated with excessive release of IL-1β, due to constitutive activation of the NLRP3 inflammasome platform; Mevalonate Kinase Deficiency, in which increased release of IL-1β is the indirect result of a metabolic defect involving the biosynthesis of sterols; and interferonopathies, which are monogenic disorders similar to Systemic Lupus Erythematosus, in which excessive levels of Type I IFNs are the main mediator of inflammation.

**Figure 1:**
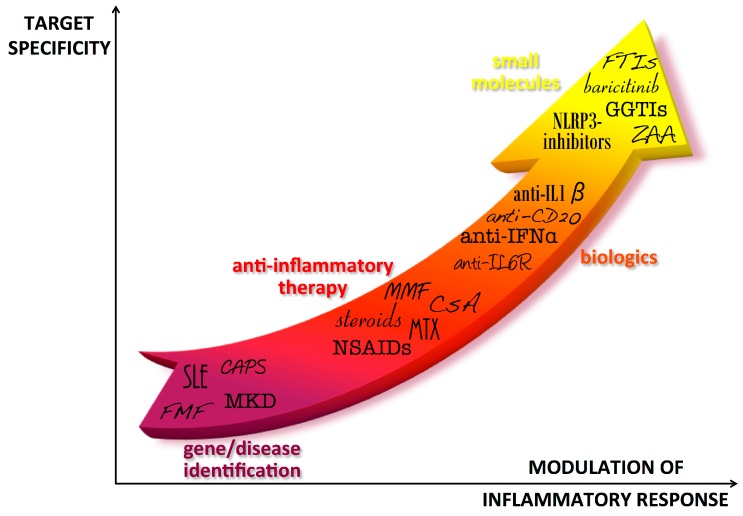
Progress in autoinflammatory disease therapy. After the diagnosis of autoinflammatory diseases, first line therapies have been characterized by treatment with anti-inflammatory drugs that generally allowed relief of symptoms but could imply severe adverse effects, especially in the long-term. The identification of the cytokines involved in the pathogenesis of the diseases was the basis for using biologic drugs directly targeted to the pathogenic cytokines. In the last years, new molecules have been developed to target specific molecules involved in the inflammatory pathways of the diseases, theoretically allowing for a better tuning of cytokine levels. IFP: IFNs-pathies; CAPS: Cryopyrin-Associated Periodic Syndromes; MKD: Mevalonate Kinase Deficiency; FMF: Familial Mediterranean Fever; NSAIDs: Nonsteroidal anti-inflammatory drugs; MTX: Methotrexate; CsA: Cyclosporin A; MMF: Mycophenolate Mofetil; IL-6R: Interleukin-6 receptor; IFN-α: Interferon-α; IL-1β: interleukin-1β; NLRP3: NLR family, nucleotide-binding domain, leucine-rich family (NLR), pyrin domain 3; ZAA: Zaragozig Acid; GGTIs: geranylgeranyl transferase inhibitors; FTIs: farnesyl transferase inhibitor.

In all cases, biological inhibitors based on recombinant proteins or antibodies have been used to control inflammation in clinical trials. More recently, the use of small molecules acting on cytokine signaling has also been proposed for these disorders (Figure 1).

We here discuss the potential and the theoretical limits of novel therapeutic strategies based on small molecules.

## 2. Cryopyrin-Associated Periodic Syndromes

Cryopyrin-associated periodic syndromes (CAPS) are a group of rare, monogenic autoinflammatory diseases arising from mutations in the Cryopyrin gene.

Depending on the type of mutation, three subtypes of CAPS can be identified, reflecting different degrees of severity:

- Familial Cold Autoinflammatory Syndrome (FCAS) (Online Mendelian Inheritance in Man, OMIM #120100);
- Muckle-Wells Syndrome (MWS) (OMIM #191900);
- Neonatal-Onset Multisystem Inflammatory Disease (NOMID) (also called Chronic Infantile Neurologic Cutaneous Articular, or CINCA, Syndrome) (OMIM #607115).

There are some clinical characteristics that are common to all three diseases: rash, fever/chills, joint pain, eye redness/pain, and headache. Additionally, CINCA/NOMID syndrome, which is the most severe form of CAPS, is characterized by development of significant disabilities, including optic nerve abnormalities (papilledema), chronic aseptic meningitis, mental impairment, facial malformation, hearing loss, and arthropathy with aberrant ossification [5].

CAPS are caused by dominant mutations of the NLRP3 (nucleotide-binding domain, leucine-rich family (NLR), pyrin domain containing) gene, encoding cryopyrin.

This protein plays an important role in the inflammasome, an essential component of the immune system, leading to the release of inflammatory cytokine IL-1β. CAPS mutations are associated with gain of function of cryopyrin, resulting in IL-1β overproduction, which is ultimately responsible for the typical inflammatory features [6].

IL-1β is not present in cells from healthy individuals, it is not constitutively expressed, and only few kinds of cells are able to produce IL-1β, such as blood monocytes, tissue macrophages, and dendritic cells [7]. In physiologic conditions, the release of IL-1β can be induced by pathogen-associated molecular patterns (PAMPs) or by damage-associated molecular patterns (DAMPs), but also by other cytokines such as TNF, IL-18, IL-1α or by IL-1β itself. The cytokine is produced as an inactive precursor that requires intracellular cleavage by caspase 1, activated by proteins of the inflammasome such as cryopyrin [8].

### 2.1. From Anti-Inflammatory to Biological Drugs

In the past, different anti-inflammatory drugs were used in the attempt to control the symptoms of the disease, but with limited success. Non-steroidal anti-inflammatory drugs (ibuprofen or naproxen) were the first line for the treatment of the inflammatory features of CAPS, but limited efficacy and serious side effects (gastrointestinal complications and bleeding) restricted their use in clinical practice. Treatment with glucocorticoids is more effective in relieving pain and febrile episodes, but undesired adverse events such as hypertension, opportunistic infections, loss of bone and skin integrity, growth retardation, and metabolic disturbances limit the prolonged use of these drugs. In addition to steroids, immunosuppressive therapies (methotrexate, cyclosporine, azathioprine, cyclophosphamide) have been tried, but always with a poor balance between costs and benefits [5].

With the introduction of biologic agents targeting IL-1β, thus able to target the main source of inflammation, therapy has greatly improved patients’ quality of life, leading to complete and sustained remission of symptoms in almost all cases and preventing, or even rescuing, inflammatory organ damage.

There are three commercial biological anti-IL-1β drugs: anakinra, rilonacept and canakinumab.

The anakinra is a recombinant, non-glycosylated, form of human interleukin-1 receptor antagonist (IL-1Ra), an endogenous molecule that binds to the IL-1 receptor and inhibits the pro-inflammatory effects of IL-1. Anakinra results in significant relief of clinical symptoms, without the need of other immunosuppressive drugs, even if the response to treatment can only be partial in severe cases of CINCA [9].

Rilonacept is a soluble decoy receptor fusion protein (extracellular domains of IL-1 type 1 receptor and IL-1 receptor accessory protein joined to the constant region of human immunoglobulin G1) that binds soluble IL-1 (α and β), preventing activation of cell surface receptors [10]. This drug has been studied in CINCA and MWS, and leads to a significant reduction in symptoms.

Canakinumab is a human anti-IL-1β monoclonal antibody, able to induce complete remission in most patients, though severe cases may require a dose escalation. However, due to its strength, it might increase the risk of infections [11].

Nowadays, canakinumab is the best choice for the treatment of the CAPS: it does not block the IL-1α binding to its receptor, and compared to anakinra (daily dosage) and rilonacept (weekly administration), one subcutaneous injection every 4–8 weeks appears to be enough.

### 2.2. Small Molecules for CAPS

Despite these encouraging results obtained by biological agents, continuous innovation in immunomodulatory drug development is required to address some limitations of protein therapies, such as the inability to modulate intracellular proteins that regulate immune cell function [12,13,14], the functional redundancy among inflammatory cytokines, or the limited delivery of protein-based reagents to mucosal tissues [15]. In fact, anti-IL-1 treatments can have limited efficacy on some complications of the diseases like hearing loss, bone dysplasia, and mental retardation [16]. Another limitation of anti-cytokine proteins comes from their high costs and their mode of administration which often requires a specialist [17].

For all these reasons, in the last years a number of small molecules have been proposed to tune the expression or function of intracellular proteins that can give rise to aberrant cytokine signaling or that can mediate their downstream consequences. Of course, CAPS can again represent the model to test the efficacy of these novel treatments.

To regulate IL-1β production three main targets are currently under focus: caspase-1, histone deacetylase and NLRP3 [18].

VX-765 is an orally active caspase-1 inhibitor that is able to block the conversion of the IL-1β precursor into an active form of IL-1β. This molecule has been demonstrated to inhibit IL1-β secretion in LPS-stimulated cells from FCAS patients [19] and in animal models [20]. Two phase 2 trials have been carried out in patients with psoriasis (NCT00205465) and epilepsy (NCT01048255) [21], while only a small open-label study has been conducted in six patients with MWS showing partial clinical improvement [22].

Acetylation and deacetylation of histones is one of the mechanisms to modulate gene expression, resulting in binding of transcription factors to DNA. This process is regulated by two enzymes: histone acetyltransferases and histone deacetylases (HDACs) [23]. ITF2357 (*givinostat*) is a histone deacetylase inhibitor with anti-inflammatory properties. In a phase 1 clinical trial, HDAC inhibitors have been shown to be safe and well tolerated, and able to inhibit pro-inflammatory cytokines such as IL-1β, TNF-α, IFN-γ and IL-6 [24]. For this reason *givinostat* could represent a promising drug to treat CAPS. However, there are some doubts about the real selectivity of these drugs, which may influence the expression of many other genes.

Finally, inhibitors of NLRP3 have been also evaluated, whose potential is of particular interest based on the pivotal role of the NLRP3 inflammasome in inflammation. In past years, some of such drugs (glyburide, CRID3, parthenolide15, 3,4-methylenedioxy-β-nitrostyrene16, and dimethyl sulfoxide) have already been proposed and used with limited success due to poor potency and nonspecific effect [25,26,27,28,29]. In October 2015, Rebecca Coll and collaborators described a new potent, selective, small-molecule inhibitor (MCC950) able to specifically block the activation of NLRP3, but not the AIM2, NLRC4 or NLRP1 inflammasomes. In animal models of experimental autoimmune encephalomyelitis (EAE), MCC950 is able to reduce IL-1β production *in vivo* and to improve the symptoms of the disease. Moreover, this molecule prevented neonatal death in a mouse model of MWS, and was shown to block NLRP3 activation in peripheral blood mononuclear cells from MWS patients [30,31]. Thus, MCC950 could be a valuable therapeutic choice for NLRP3-associated syndromes, including autoinflammatory and autoimmune diseases, but further clinical trials are needed to better understand the potential of this small molecule.

Direct targeting of NLRP3 is of particular interest if we consider recent data showing how activated NLRP3 inflammasome, by recruiting the protein adaptor ASC, can act to propagate and amplify inflammation from cell to cell [32]. Thus, NLRP3 activation could be a better target to act on early events of inflammation before inflammatory amplification has started occurring.

Furthermore, the confirmation of the safety and efficacy of these drugs could open the way to their use for other diseases, whose course can be worsened by an IL-1β-mediated inflammatory response (gout, diabetes mellitus type 2, cortical strokes, and following myocardial infarction), as already done with the biological agents [33,34,35,36,37,38].

## 3. Mevalonate Kinase Deficiency

Mevalonate Kinase Deficiency (OMIM #260920; MKD) is a rare and neglected disease, due to mutations in the mevalonate kinase gene (MVK) coding for mevalonate kinase (MK), an enzyme of the mevalonate pathway for the biosynthesis of cholesterol and non-sterol isoprenes [39,40]. The residual activity of MK defines different degrees of MKD severity, ranging from an auto-inflammatory phenotype (Hyper IgD Syndrome/HIDS; OMIM #260920), to a very severe clinical presentation (mevalonic aciduria/MA; OMIM #610377) [41]. The phenotype of HIDS typically includes only recurrent episodes of fever and associated inflammatory symptoms such as oral ulcers, skin rashes, arthralgia, abdominal pain, and diarrhea. Patients with MA show, in addition to these episodes, developmental delay, dysmorphic features, ataxia, cerebellar atrophy, psychomotor retardation and may die in early childhood [42,43,44].

To date, the pathogenesis of MKD is still a matter of study, in particular as concerns the neurological involvement.

The study of MA pathogenesis is quite difficult because the only existing murine model of the disease is created with a heterozygous knock-out deletion of the MKV gene [45], resulting in a mild disease phenotype, lacking the features of neurological dysfunction. Complete shortage of other enzymes in the same pathway, upstream [46] or downstream [47] MK in mice have revealed a high degree of embryonic lethality. Moreover, cell lines from MA patients do not exist: the anatomical evaluations about neurological impairment of MKD can only be done post-mortem. The only alternative, so far, has been provided by cell lines treated with biochemical inhibitors to produce a deficiency in the mevalonate pathway. Although these models did not reproduce the same defect observed in MA, they could shed some light on biochemical mechanisms relevant to the disorder [48,49].

### 3.1. Biological Drugs for MKD

MKD is an orphan disease and the current treatment options are mainly targeted at relieving inflammatory symptoms [50]. While anti-inflammatory drugs and on demand steroids provide acceptable control of symptoms in patients with milder forms of the disease, lifelong treatment with biological drugs (such as anakinra or canakinumab) is usually required for patients with high recurrence of severe inflammatory attacks [51,52]. Furthermore, the only valuable therapeutic option for patients with MA is hematopoietic stem cell transplantation which, however, is burdened with a series of risks and complications [53].

### 3.2. Small Molecules for MKD: Inhibitors of Mevalonate Pathway

Recent literature data showed that several molecules, as farnesyl transferase inhibitors (FTIs), geranylgeranyltransferase inhibitors (GGTIs), isoprenoids, or squalene synthase inhibitors, acting on the mevalonate pathway are able to modulate the inflammatory response.

FTIs (tipifarnib and lonafarnib) and GGTIs are two classes of molecules acting on post-translational modifications of Ras proteins, and can have opposite effects on the modulation of inflammation. In particular, FTIs have been shown to have an anti-inflammatory action, which could be mediated both by reduced farnesylation of Ras proteins and by diversion of mevalonate derived isoprenoids toward geranylgeranylation of Rac1 and RhoA GTPases. In contrast, GGTIs were demonstrated to be able to increase the inflammatory response. However, preclinical data are still insufficient to justify the experimental use of these drugs in clinical trials in patients with MKD.

Furthermore, preliminary investigations have shown a potential synergy between FTIs and mevalonate-derived isoprenoids such as geranylgeraniol both on MKD cellular models and on patients’ samples [54]. Other bioactive isoprenoids with a potential in MKD include geraniol, farnesol, limonene and menthol.

Of note, the mevalonate pathway is the focus of pharmacological approaches for different medical conditions, from osteoporosis, to cancer and lipid metabolism. The different effects of drugs active on this pathway are not always easily predictable, due to the complex regulation of enzymes in the mevalonate pathway.

Aminobisphosphonates act on different enzymes downstream of mevalonate, reducing the availability of isoprenoid intermediates in a variable manner, leading to various biological effects, including inhibition of bone resorption and tumor proliferation. Aminobisphosphonates have been used to induce an MKD-like inflammatory reactivity *in vitro* and in animal models [55,56].

Statins are cholesterol lowering agents acting upstream of the mevalonate pathway by reducing the availability of mevalonate and the production of endogenous cholesterol. They are used in the pharmacological treatment of diseases such as hypercholesterolemia, atherosclerosis, and cardiovascular disease [57].

To avoid undesired effects of statins, which have been related to a shortage of cholesterol derived isoprenoids, other cholesterol lowering agents have been proposed, inhibiting squalene synthase, the enzyme responsible for the synthesis of sterols from isoprenoids. Several squalene synthase inhibitors have been developed, such as zaragozic acids or 2,8-dioxabicyclo[3.2.1]octane derivatives, dicarboxylic acid and quinuclidine derivatives, 4,1-benzoxazepine and substituted morpholine derivatives [58]. Zaragozic acid, in particular, reversed the defect in geranylgeranylation of RhoA and Rac1 in cells from patients with MA [59] and displayed an appreciable anti-inflammatory effect on cells from patients with MKD [60]. Although some of these drugs, such as lapaquistat, have shown a good safety profile in volunteers, no clinical trials have yet been performed in MKD [61].

## 4. Interferonopathies

Interferonopathies are a particular category of autoinflammatory diseases [62], grouping few rare disorders characterized by highly deregulated production of class I IFNs [63,64]. These disorders arise from defects in the pathway of sensing of nucleic acids in the cells, and include deficiency of DNAses, RNAses and gain of function mutations of the adaptor protein STING (Stimulator of Interferon Genes) [65,66]. Clinical features show significant overlap with Systemic Lupus Erythematous (SLE) (OMIM #152700), which is as well associated with increased signaling by class I IFNs. Indeed, anti-double-stranded DNA auto-antibodies (anti-dsDNA) are found both in SLE and in interferonopathies and can be the results of a defective disposal of nucleic acids derived from apoptotic cells [67,68]. However, while in sporadic SLE autoimmune features are usually the prominent features, interferonopathies are characterized by more severe inflammation, poor response to conventional treatments and worse outcomes.

### 4.1. From Monogenic Interferonopathies to SLE

A critical issue to be solved is whether knowledge on monogenic causes of inflammatory disorders may open the way to the identification of molecular targets for therapy and to the development of novel treatments. This goal is highly relevant in pediatrics, given the chronic and severe course of early onset forms of SLE, despite currently available therapies [69,70,71].

As concern the pathogenesis of SLE, the break of the normal tolerance between T- and B-cells occurring in SLE implies an altered T-cell response that, in turn, primes the B-lymphocytes to produce high-affinity auto-antibodies, which cause tissue damage. Moreover, the interaction between T- and B-cells is strictly regulated by the presence and interaction of co-stimulatory molecules (CD40/CD40L and CD28/B7) and secretion of inflammatory cytokines, such as TNF-α and IL-6 [72]. Every step of this inflammatory pathway is a conceivable target for SLE therapy. In fact, in the past, the disease was treated with immunosuppressive drugs (*i.e.*, corticosteroids, cyclosporin, mycophenolate mofetil, cyclophosphamide, azathioprine), that allowed an improvement in the course of the disease, but that were burdened by serious adverse effects, especially in the long term [73,74]. During the past years more specific treatments were developed against one or more of the steps involved in the inflammatory process mentioned above. Even though the possible targeted therapies were investigated in different clinical trials, their substantial efficacy is still unclear because of the difficulty of conducting randomized controlled trials, due to the heterogeneity of the disease itself. Thus, to date, none of these biologics have an established use in clinical practice for the treatment of SLE, with the exception for Rituximab, a monoclonal antibody to the CD20 antigen expressed on B cells, and Belimumab, a monoclonal antibody to B-cell activating factor (BAFF), that have demonstrated improvements in controlling the worst manifestation of SLE, the lupus nephritis [75]. The first remains the treatment of choice in case of SLE refractory to conventional treatments [76,77], while the second is the only biologic approved for SLE that demonstrated improvements in antibody titers and disease activity [78,79,80].

The recent identification of a group of monogenic interferonopathies may shed some light on the pathogenesis of SLE and may help resolve open issues concerning resistance to conventional treatments in some cases, in particular among those with early onset in childhood. In fact, deficiencies of TREX1 and RNASEH2C are responsible for rare disorders that were considered as familial forms of SLE (Chilblain Lupus; Aicardi Goutieres syndrome). Recent studies demonstrated that a key molecule in these inflammatory disorders is the adaptor protein STING. These results are in agreement with the recent identification of the SAVI syndrome, (STING-associated vasculopathy, infantile onset), a monogenic disorder due to activating mutations of STING, which shares significant pathological features with other interferonopathies (OMIM #615934) [81].

Indeed, the identification of high levels of type I IFNs, and in particular of IFN-α, and the observation of a set of inflammatory genes induced by IFNs in biologic samples from patients with SLE (interferon signature), have demonstrated a common pathogenic pathway that underlies the heterogeneous spectrum of the disease and can also explain the onset of lupus-like syndromes or other condition with anti-dsDNA negative sera [82,83,84,85]. Type I IFNs were produced in response to viral nucleic acids, but also in response to endogenous DNA recognized by cellular sensors such as TLR7, TLR9 and cGAS, mainly through the activation of STING. In particular, endogenous DNA is released during apoptosis and necrosis of cells and can induce the breakdown of T- and B-cell immune tolerance, especially in presence of concomitant genetic alteration that predispose to SLE, since in SLE patients the clearance of cell debris after apoptosis and necrosis is impaired [86,87].

All this considered, an interesting therapeutic strategy is the blockade of the IFN pathway by inhibiting IFN-α by administering a monoclonal antibody. The most studied monoclonal antibodies anti-IFN-α are Sifalimumab, an IgG1 human monoclonal antibody that binds to IFN-α and prevents the signaling through its receptor [88,89,90], and Rontalizumab, an IgG1 monoclonal antibody with inhibitory activities on multiple isoforms of IFN-α [91,92].

### 4.2. Small Molecules in Interferonopathies

The study of the SAVI syndrome highly contributed to unravel the common pathway of IFN activation in interferonopathies and may serve as a model to improve knowledge and to develop novel treatments, as well as for multigenic disorders such as SLE, which can be associated with deregulated IFN signature.

Activation of STING leads to enhanced transcription of IFN regulating genes and to strongly-increased production of type I IFNs. In turn, IFNs exert their inflammatory action though binding of the receptor IFNAR1/2 on the surface of cells and leading to JAK1/TYK2 and STAT1/2 activation and downstream transcription of IFN-dependent pro-inflammatory mediators [93,94].

In very recent years, small molecules were developed to interfere with the inflammatory pathways, depending on cytokine signaling. Different molecules that act as JAK1/JAK2 inhibitors underwent evaluation in clinical trials [95]. Among them, Ruxolitinib was already approved by FDA for the treatment of myelofibrosis and has also demonstrated significant improving in rheumatoid arthritis [96,97], and Baricitinib, which is currently under evaluation in multiple clinical trials, in particular for the treatment of rheumatoid arthritis [98]. Of note, blocking JAK signaling with Baricitinib was shown to be able to reduce the inflammatory response in cells from subjects with activated STING disease [81].

Based on these data, it may be worth evaluating the potential of JAK inhibitors also in subjects with SLE who failed to respond to conventional treatments. The advantages of treatment with small molecules if compared to biologics, reside in their lower cost and in their availability for oral administration. On the contrary, the limitation in use derives obviously from their recent access as a therapeutic tools, and consequently to poor knowledge in particular of long-term efficacy and safety and possible adverse effects both for prolonged therapy and for discontinuation [99,100,101].

## 5. Conclusions

After four decades since the first development of monoclonal antibodies, the promise of more selective and safe therapies has been kept. The market of therapeutic antibodies and other recombinant proteins has shown a constant increasing trend. Although most prescriptions relate to malignancies and multifactorial inflammatory diseases, the best demonstrations of efficacy have been obtained in few rare monogenic diseases characterized by prevalent deregulation of single cytokines. On one hand, treatment with biological drugs allowed to obtain a more complete suppression of a given cytokine with lower rates of undesired effects if compared with traditional anti-inflammatory and immunosuppressive drugs. On the other hand, anti-cytokine antibodies are not free from costs and risks. Indeed, the preparation of large amount of recombinant proteins for therapy has high costs and the strong inhibitory activity is not easily adjustable. In fact, the long half-life of most monoclonal antibodies can be both an advantage and a reason of concern, in particular in case of infections.

In recent years, improved experimental settings have allowed selecting and producing increasing numbers of small molecules with definite molecular targets. When compared with protein-made-biologicals, these novel drugs may present advantages for the ease of administration and for a more precise tuning of their action, and in many cases for their lower production costs. In addition, small molecules can have a better distribution in diseased organs and cells. The development of drugs acting on definite molecular pathways can allow the inhibition of more than one target cytokine with the same drug. In several cases, small inhibitors developed to treat tumors may come in handy to treat inflammatory diseases whose pathogenesis involves the same molecular pathways. Compared with cytokine blocking agents, small molecules may have the advantage of acting on earlier pathogenic events. Again, rare monogenic disorders can provide valuable models for the evaluation of these novel drugs. In particular cases, such as MKD, the study of the molecular mechanisms of the diseases can help find novel targets for therapy. Great caution should be taken in considering the possibility that novel small molecules may have unpredicted effects in addition to their action on the selected molecular target. However, given the increasing knowledge on the molecular mechanisms of genetic disorders, it is predictable that several inhibitors will find a place, in the near future, in the so called “precision medicine”.
